# A Retrospective Unicenter Study of Clinical and Inflammatory Features in Hospitalized Adults with Respiratory Syncytial Virus Infection Across Two Epidemic Waves in Catalonia, Spain

**DOI:** 10.3390/jcm15114184

**Published:** 2026-05-28

**Authors:** Simona Iftimie, Julia Fambuena-González, Andrea Jiménez-Franco, Joaquín Fernández-López, Eva María Declara-Declara, Ana Felisa López-Azcona, Xavier Gabaldó-Barrios, Jordi Camps, Antoni Castro

**Affiliations:** 1 Autoimmunity, Infection and Thrombosis Research Group (GRAIIT), Department of Internal Medicine, Hospital Universitari de Sant Joan, Institut de Recerca Biomèdica Catalunya Sud, Universitat Rovira i Virgili, Av. Dr. Josep Laporte 2, 43204 Reus, Spain; simona-mihaela@salutsantjoan.cat (S.I.); julia.fambuena@estudiants.urv.cat (J.F.-G.); eva.declara@salutsantjoan.cat (E.M.D.-D.); anafelisa.lopez@salutsantjoan.cat (A.F.L.-A.); antoni.castro@urv.cat (A.C.); 2 Unitat de Recerca Biomèdica, Hospital Universitari de Sant Joan, Institut de Recerca Biomèdica Catalunya Sud, Universitat Rovira i Virgili, 43204 Reus, Spain; andrea.jimenez@urv.cat; 3 Department of Clinical Laboratory, Hospital Universitari de Sant Joan, Institut de Recerca Biomèdica Catalunya Sud, Universitat Rovira i Virgili, 43204 Reus, Spain; joaquin.fernandez@salutsantjoan.cat (J.F.-L.); xavier.gabaldo@salutsantjoan.cat (X.G.-B.)

**Keywords:** biomarkers, C-reactive protein, interleukin-6, lower respiratory tract infection, neutrophil–lymphocyte ratio, respiratory syncytial virus, systemic inflammation

## Abstract

**Background**: Respiratory syncytial virus (RSV) is a serious disease in older adults and is associated with various comorbidities; however, comparative data across epidemic waves, both clinically and in terms of inflammatory profiles and their diagnostic and prognostic utility, remain limited. **Methods**: We conducted a retrospective study of adults hospitalized with RSV infection across two epidemic waves (2022–2023 and 2024–2025). Data on clinical characteristics, comorbidities, severity scores, and outcomes were collected, and serum interleukin-6 (IL-6), C-reactive protein (CRP), and hematological parameters were analyzed and compared with those in healthy controls. **Results**: A total of 152 patients were included in this study (81 in wave 1 and 71 in wave 2). Patients in wave 2 were older and had a higher burden of comorbidities, although ICU admission and in-hospital mortality were similar across waves. RSV induced a consistent systemic inflammatory response in both waves, characterized by elevated IL-6 and CRP levels, neutrophilia, lymphopenia, and increased neutrophil-to-lymphocyte ratios, with no relevant inter-wave differences. All biomarkers demonstrated good diagnostic performance. The neutrophil-to-lymphocyte ratio showed the highest accuracy, while IL-6 exhibited high rule-in capacity. However, none of the evaluated biomarkers were associated with disease severity or mortality. **Conclusions**: RSV infection in older adults is associated with a similar inflammatory profile across waves. Although biomarkers showed strong diagnostic utility, they did not show any significant prognostic discrimination in this cohort. We suggest that disease severity is primarily associated with host-related factors, particularly comorbidities, rather than with differences in the inflammatory response, highlighting the need for improved preventive and risk-stratification strategies in this population.

## 1. Introduction

Respiratory viral infections represent a major cause of morbidity and hospitalization among adults, particularly in older individuals and those with chronic comorbidities. Among these pathogens, respiratory syncytial virus (RSV) has traditionally been regarded as a pediatric pathogen. However, increasing evidence over the past decade has demonstrated its substantial clinical impact in adults [1,2,3]. In older populations, RSV has been estimated to account for 3–7% of hospitalizations due to acute respiratory infections, with tens of thousands of hospital admissions annually in high-income countries [4]. Clinical outcomes can range from mild upper respiratory symptoms to pneumonia, respiratory failure, and death, particularly in patients with chronic pulmonary or cardiovascular comorbidities [5,6].

Epidemiology and clinical presentation of RSV infection may vary significantly between epidemic seasons. Variations in viral circulation patterns, the emergence of different RSV subtypes, and fluctuations in population immunity can influence both hospitalization incidence and disease severity. In addition, healthcare practices, such as changes in testing strategies, vaccination coverage for other respiratory pathogens, and hospital admission policies, may further impact which patients are identified and admitted during each season [7]. Moreover, disruptions caused by the global SARS-CoV-2 pandemic have been associated with profound alterations in the timing, magnitude, and age distribution of seasonal RSV outbreaks [8]. Some reports have documented shifts in peak incidence, higher proportions of adult hospitalizations, and differences in clinical severity compared to pre-pandemic seasons, suggesting that both viral and host factors may have evolved [9]. Despite the well-recognized impact of RSV in adults and its comparable hospital-related disease burden to that of other respiratory viruses, detailed comparisons of adult cohorts across different epidemic waves are lacking, particularly regarding clinical characteristics and outcomes between successive seasons [10]. Understanding these seasonal differences is essential for anticipating healthcare needs, identifying patients at higher risk of severe disease, and guiding the development of targeted preventive and therapeutic strategies.

The identification of clinically accessible biomarkers that could help to characterize the inflammatory response associated with RSV infection in adults represents an emerging area of research. This disease is known to activate the innate immune system through epithelial and immune cell recognition, leading to the release of pro-inflammatory cytokines and the subsequent development of a systemic inflammatory response. This host response, characterized by acute-phase activation, is thought to contribute to both local respiratory pathology and systemic manifestations of the disease. Among the mediators involved, interleukin-6 (IL-6) plays a central role in the acute-phase response, while C-reactive protein (CRP) reflects downstream hepatic activation. Both are widely used inflammatory markers that capture different aspects of the host response to infection [11,12]. Evaluating these routinely available parameters in hospitalized patients may contribute to better descriptive characterization of respiratory syncytial virus infection across different epidemic periods in a real-world clinical setting.

In this context, we conducted a single-center observational study of adults hospitalized with RSV infection during two epidemic waves (2022–2023 and 2024–2025). We compared demographic and clinical characteristics, severity-related clinical variables, and routinely available inflammatory parameters, including IL-6 and CRP, with the aim of providing a descriptive overview of patient profiles, inflammatory response patterns, and clinical outcomes across two epidemic periods in a specific geographical area.

## 2. Materials and Methods

### 2.1. Study Design

We conducted a retrospective observational study in hospitalized patients with RSV infection at Hospital Universitari de Sant Joan de Reus, located in the Autonomous Community of Catalonia, Spain. We included a total of 152 patients admitted between 1 October 2022 and 30 April 2023 (*n* = 81) and between 1 October 2024 and 30 April 2025 (*n* = 71). Our facility comprises a general hospital with 367 inpatient beds and an intensive care unit with 20 beds, and provides healthcare coverage for a population of more than 175,000 inhabitants, including patients from primary care centers and long-term care facilities in the surrounding region.

The inclusion criteria were an age ≥ 18 years and attendance at the Emergency Department with laboratory-confirmed RSV infection. The study included both patients who required hospital admission and those who were evaluated in the Emergency Department and discharged home. Patients without laboratory confirmation of RSV infection were excluded. Demographic and clinical characteristics were recorded for all patients, including presenting symptoms, comorbidities, and treatments. Disease severity was assessed using the McCabe score [13], and RSV infection was confirmed using the Xpert^®^ Xpress Flu/RSV assay (Cepheid, Sunnyvale, CA, USA), a rapid real-time reverse transcription polymerase chain reaction test performed using the GeneXpert System. The assay was carried out on nasopharyngeal swab specimens collected in viral transport medium, and viral positivity was defined according to the manufacturer’s instructions. IL-6 concentrations were measured via an Elecsys^®^ IL-6 electrochemiluminescence immunoassay using a Cobas e801 analyzer (Roche Diagnostics, Basel, Switzerland), with analytical sensitivity and precision established according to the CLSI EP17-A2 and EP05-A3 guidelines, and calibration traceable to the NIBSC IL-6 international standard. CRP was determined via a particle-enhanced immunoturbidimetric assay (Tina-quant^®^ CRP IV) using a Cobas c702 analyzer, with calibration traceable to the ERM-DA474/IFCC reference material. Leukocyte and neutrophil counts were obtained using a Sysmex XN-1000 (Sysmex Corp., Kobe, Japan) automated hematology analyzer based on fluorescence flow cytometry principles. All assays were subjected to routine multi-level internal quality control procedures and regular calibration according to standard laboratory practice. The clinical laboratory is accredited by the ISO 15189 standard for medical laboratories. The biomarkers included in the analysis were selected based on their routine availability in the clinical management of patients with suspected respiratory infections and their consistent documentation in the electronic medical records during the study period. Specifically, IL-6, CRP, and standard hematological indices were systematically available for all included patients as part of routine laboratory assessment, whereas other inflammatory mediators were not routinely measured in clinical practice and were therefore not available in the retrospective dataset.

Analytical determinations were compared using a control group of 80 healthy volunteers with no clinical or biochemical evidence of infectious disease, renal insufficiency, liver disease, neoplasia, or neurological disorders. The samples were obtained before the COVID-19 pandemic from a study conducted on a healthy population by the epidemiology department of our university. Participants were recruited through telephone interviews based on census data from several municipalities in the region, and each participant subsequently underwent a clinical interview and basic laboratory testing [14]. In this cohort, hematological parameters were measured in fresh blood samples at the time of collection, and serum samples were processed according to standard laboratory procedures and analyzed for biochemical parameters within the framework of the original study.

All data were obtained from medical records and were fully anonymized before the researchers accessed them. This study was approved by the Comitè d’Ètica i Investigació en Medicaments (Institutional Review Board) of Institut de Recerca Biomèdica Catalunya Sud (Resolution CEIM 344/2025, 26 March 2026).

### 2.2. Statistical Analysis and Effect Size Estimation

Qualitative data are presented as numbers and percentages; age and duration of hospital and intensive care unit stay are shown as medians and ranges (minimum–maximum) due to their highly skewed distribution and the presence of extreme values; and analytical variables are presented as medians and interquartile ranges (IQRs). Statistical comparisons between two groups were made using the χ^2^ test (categorical variables) or the Mann–Whitney U test (continuous variables), while the diagnostic accuracy of the analyzed variables was assessed through receiver operating characteristic (ROC) analysis [15]. To assess the robustness of the estimates, internal validation was performed using bootstrap resampling (2000 iterations), and statistical comparisons between areas under the curve (AUCs) were performed using the DeLong test. The sensitivity, specificity, positive predictive value (PPV), and negative predictive value (NPV) were calculated for each biomarker, and optimal cut-off values were determined by maximizing the Youden index. Effect sizes were calculated as rank-biserial correlation (r) for continuous variables, together with Hodges–Lehmann median differences (wave 1–wave 2) and 95% confidence intervals. For categorical variables, effect sizes were expressed as odds ratios (ORs) with 95% confidence intervals for binary variables, and Cramér’s V for variables with more than two categories. We employed SPSS 25.0 (SPSS Inc., Chicago, IL, USA) and R 4.3.0 for statistical analyses. All data were visualized using ggplot2, gridExtra, and pROC in R. Statistical significance was set at *p* ≤ 0.05.

## 3. Results

The demographic and clinical characteristics of patients from the two infection waves are summarized in Table 1. Patients in the second wave were older than those in the first, and although most patients in both waves were admitted to the Internal Medicine department, admissions to Geriatrics were more frequent during the second wave. In addition, these patients were more commonly affected with pneumonia and bronchitis and had a higher prevalence of cardiovascular diseases. While no differences were observed in the Charlson comorbidity index, patients in the second wave showed greater clinical severity according to the McCabe classification. They also more frequently required non-invasive mechanical ventilation and were more often treated with corticosteroids, whereas anticoagulant use was less common in these patients. Overall, these findings suggest a shift toward a more clinically complex and vulnerable patient population. Despite these results, no significant differences were found between the two waves in terms of mortality, intensive care unit (ICU) admission rates, ICU length of stay, or overall hospital length of stay.

**Figure 1:**
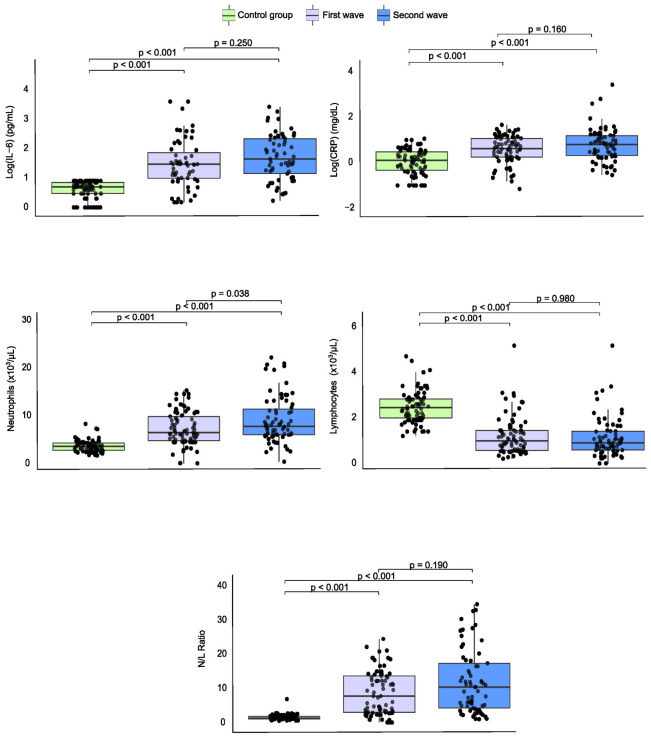
Distribution of laboratory variables in patients with respiratory syncytial virus infection across the first and second epidemic waves and healthy controls. Data are presented as medians and interquartile ranges. The sample sizes for each group are as follows: control group, *n* = 84; first wave, *n* = 81; second wave, *n* = 71. Group comparisons were performed using unadjusted Mann–Whitney U tests. The exact *p* values are displayed in the figure. IL-6 and CRP are shown as log10-transformed variables due to skewed distributions. Abbreviations: CRP, C-reactive protein; IL-6, interleukin-6; N/L ratio, neutrophil-to-lymphocyte ratio.

The results of the analysis of variables across the two epidemic waves and the control group are shown in Figure 1. Compared with healthy individuals, patients with RSV infection exhibited significantly higher levels of IL-6 and CRP, as well as increased neutrophil counts. In contrast, their lymphocyte counts were lower, resulting in an elevated neutrophil-to-lymphocyte (N/L) ratio. Patients in the second epidemic wave showed slightly but significantly higher neutrophil counts than those in the first wave. No other significant differences were observed between the two waves.

In addition to *p* values, effect size estimates (Wilcoxon r) were calculated, and Hodges–Lehmann median differences with 95% confidence intervals were also computed to provide a clinically interpretable measure of group differences. Effect sizes were small or negligible across variables, and confidence intervals overlapped zero for most biomarkers, indicating limited clinical relevance. A small but statistically significant difference in neutrophil counts was observed between epidemic waves (Table 2).

**Figure 2:**
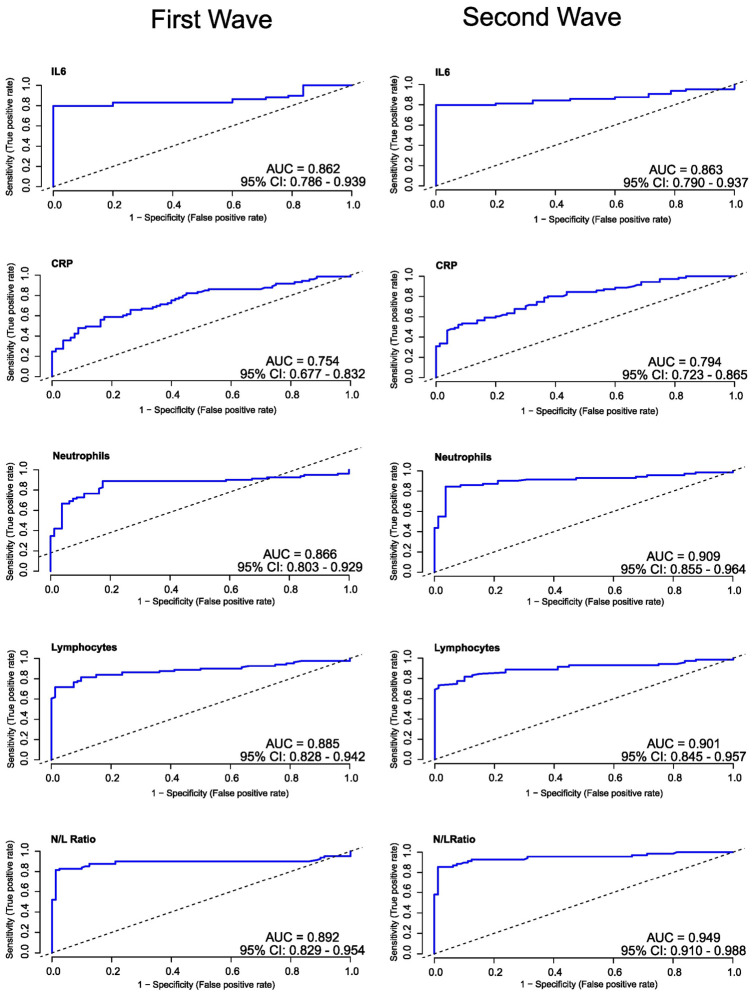
Receiver operating characteristic curves for the diagnostic performance of all analyzed parameters in respiratory syncytial virus-infected patients versus healthy controls. Abbreviations: AUC, area under the curve; CRP, C-reactive protein; CI, confidence interval; IL-6, interleukin-6; N/L ratio, neutrophil-to-lymphocyte ratio.

To evaluate diagnostic performance, we assessed the ability of the analyzed variables to discriminate between RSV-infected patients and healthy controls using ROC curve analysis. These analyses were first performed separately for each epidemic wave to assess the consistency of the diagnostic performance of the studied biomarkers across both periods. Most parameters showed strong discriminative capacity, with AUCs exceeding 0.85 for all variables except CRP (Figure 2).

Bootstrap resampling (2000 iterations) confirmed the stability of the AUC estimates, with confidence intervals consistent with those obtained in the primary analysis (Table 3).

Comparisons of AUCs between the first and second epidemic waves using the DeLong test showed no statistically significant differences across biomarkers (Table 4).

As no relevant differences in ROC patterns were observed between waves, the data were subsequently pooled to calculate sensitivity, specificity, PPV, and NPV. The diagnostic performance of the evaluated biomarkers is summarized in Table 5. The neutrophil-to-lymphocyte ratio showed the best global diagnostic accuracy among all variables assessed, with a consistently balanced ability to discriminate between RSV-infected patients and controls. Among individual biomarkers, IL-6 showed a high specificity and PPV in this cohort, although with more limited sensitivity. Neutrophils and lymphocytes also performed well overall, whereas CRP showed the lowest diagnostic accuracy.

**Figure 3:**
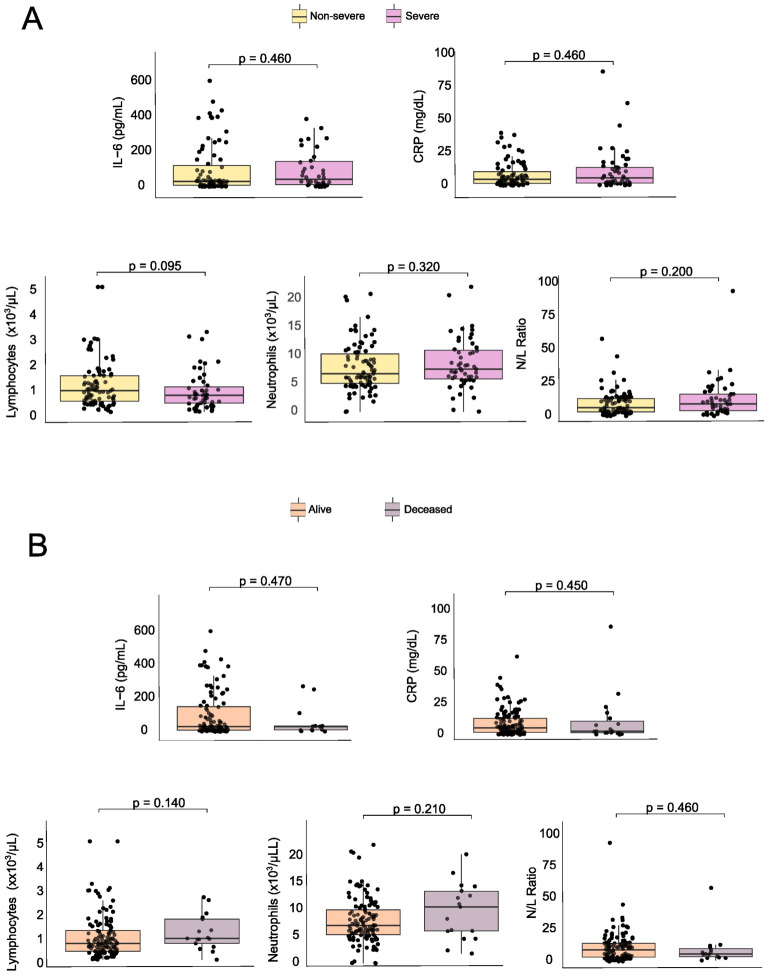
Distribution of laboratory variables in respiratory syncytial virus-infected patients according to McCabe-defined severity (**A**) and in-hospital mortality (**B**). Results are shown as medians and interquartile ranges. The sample sizes for each group are as follows: non-severe, *n* = 97; severe, *n* = 54; alive, *n* = 133; deceased, *n* = 19. Statistical significance was assessed using the unadjusted Mann–Whitney U test. Exact *p* values are displayed in the figure. McCabe indices were dichotomized into non-severe (0–1) and severe (2–3). Abbreviations: AUC, area under the curve; CRP, C-reactive protein; CI, confidence interval; IL-6, interleukin-6; N/L ratio, neutrophil-to-lymphocyte ratio.

We also explored the potential prognostic value of the analyzed biomarkers. For this purpose, we assessed their association with disease severity (using the McCabe index, dichotomized into non-severe (0–1) and severe (2–3) groups) and with in-hospital mortality. No significant differences were observed in any of the evaluated parameters across these outcomes, suggesting that, unlike their diagnostic performance, the analyzed biomarkers did not show any prognostic discrimination in this cohort (Figure 3 and Table 6).

## 4. Discussion

In this single-center retrospective study, we characterized the clinical and inflammatory features of adults hospitalized with laboratory-confirmed RSV infection across two epidemic waves (2022–2023 and 2024–2025). Our results provide a real-world overview of RSV-related hospitalizations and illustrate both stability and modest variability in patient profiles over time.

Overall, RSV infection in hospitalized adults was associated with a significant clinical impact, characterized by frequent respiratory compromise, a high prevalence of comorbidities, and substantial use of healthcare resources, including oxygen therapy and non-invasive ventilation. Patients in the second wave were older and more often managed in geriatric units, in keeping with previous reports describing the increasing relevance of RSV in older and clinically vulnerable populations [9,16,17,18,19].

Despite these differences, major severity indicators, including ICU admission, length of stay, and in-hospital mortality, remained unchanged between epidemic waves. These findings align with prior studies reporting relatively stable outcomes of RSV infection in hospitalized adults across seasons [20,21]. Although the second wave was characterized by a higher burden of comorbidity and more frequent respiratory failure, this did not translate into worse outcomes, suggesting that disease severity in hospitalized patients may be more closely related to host factors such as age and comorbidity profile, and may also be influenced by improvements in clinical management and supportive care [20,22].

From a laboratory perspective, RSV infection triggered a clear systemic inflammatory response, characterized by elevated IL-6 and CRP levels, neutrophilia, lymphopenia, and an increased N/L ratio. These findings are consistent with the current understanding of RSV immunopathogenesis, in which viral recognition by airway epithelial cells and innate immune receptors induces the release of pro-inflammatory cytokines [23,24], leading to recruitment and activation of neutrophils and amplification of systemic inflammation [25]. This neutrophil-dominated response has been associated with disease severity in RSV infection, whereas lymphopenia reflects a transient redistribution and suppression of adaptive immune responses during acute infection [25,26]. Importantly, host factors such as age, comorbidities, and baseline immune status are known to modulate the magnitude and clinical consequences of this inflammatory response [27]. The reported patterns were comparable across both epidemic waves, indicating a comparable inflammatory response between waves over time. Most evaluated biomarkers demonstrated strong diagnostic performance in distinguishing infected patients from healthy controls, supporting their usefulness as indicators of acute viral infection.

Among the evaluated parameters, the N/L ratio emerged as the most robust and balanced marker, supporting its potential utility as a primary diagnostic tool. Moreover, IL-6 showed high specificity and PPV in this cohort, suggesting promising confirmatory performance. Neutrophils also showed strong diagnostic value as an individual parameter, whereas CRP consistently performed less well, in line with its known lack of specificity as a general inflammatory marker. These findings support the added value of integrated hematological indices over isolated inflammatory biomarkers in clinical practice.

In addition to their diagnostic performance, we evaluated the potential prognostic value of the studied biomarkers. However, these parameters did not exhibit a significant association with disease severity according to the McCabe index, or with in-hospital mortality. These findings suggest that, although useful for diagnostic purposes, these biomarkers may have limited predictive value in this setting.

Overall, our findings suggest that the analyzed biomarkers primarily reflect acute systemic inflammatory activation rather than serving as determinants of disease trajectory. These observations agree with our previous work and other published studies, which have shown that IL-6 elevations reflect a generalized inflammatory response but have limited specificity for distinguishing disease entities or predicting clinical outcomes in this setting [11]. In this context, IL-6 behaves as a marker of systemic inflammation rather than a specific indicator of RSV infection.

While CRP and standard hematological indices have shown inconsistent prognostic value in RSV and other viral respiratory infections in adults [11,28], pediatric studies have suggested a potential association between IL-6 and disease severity in RSV bronchiolitis [29,30]. In contrast, our findings in older adults do not support a prognostic role for IL-6, consistent with previous studies in elderly populations that have shown elevated levels in more severe cases but substantial overlap between groups [31]. Despite its favorable diagnostic performance and high specificity in the present study, IL-6 did not translate into meaningful prognostic discrimination, highlighting the limited utility of systemic inflammatory markers for outcome prediction in this clinical setting. Although IL-6 showed high specificity in our cohort, these findings should be interpreted with caution given the absence of external validation and the lack of comparison with other acute respiratory infections or inflammatory conditions that may increase IL-6 concentrations. Nevertheless, the absence of statistically significant differences should also be interpreted with caution, since it may be partly explained by limited statistical power arising from the study’s small sample size. Therefore, a potential type II error cannot be excluded.

In the present study, we did not aim to identify novel biomarkers or explore mechanistic aspects of RSV pathophysiology, but rather to assess whether routinely available inflammatory parameters could provide clinically relevant information across different epidemic waves in a real-world hospital setting. Our results reinforce several key points. First, RSV remains an important cause of hospitalization in older adults. Second, clinical and inflammatory profiles were comparable across epidemic waves in a single-center setting. At the biological level, inflammatory biomarkers showed strong diagnostic performance overall, but limited prognostic utility. Accordingly, the poor prognostic discrimination observed with these markers underscores the need for improved risk-stratification tools that integrate host vulnerability and immune response, rather than relying on isolated inflammatory markers.

The comparison with healthy controls was intended to contextualize the magnitude of systemic inflammatory activation in RSV infection rather than to reflect a real-world diagnostic scenario involving multiple respiratory pathogens.

Effect size analyses provided additional insight into the magnitude of the observed between-wave differences beyond statistical significance. These analyses indicated that inter-wave variability was primarily driven by differences in baseline clinical characteristics, particularly comorbidity burden (as reflected by McCabe classification), which showed a large effect size. In contrast, several symptom-related variables showed small-to-moderate effects, suggesting heterogeneous but overall limited differences in clinical presentation between epidemic waves.

It should be noted that systemic inflammatory biomarkers demonstrated negligible effect sizes when comparing the two waves, with confidence intervals indicating no relevant differences. This finding suggests a limited biological divergence in the systemic inflammatory response across epidemic waves in our cohort.

Overall, these results support the interpretation that the observed differences between epidemic waves are primarily related to patient baseline vulnerability rather than substantial differences in the underlying inflammatory response to RSV infection.

Importantly, preventive strategies are becoming increasingly relevant, with RSV vaccines recently approved for use in older adults and high-risk populations. In the United States, the CDC recommends a single dose for adults aged ≥75 years and for higher-risk individuals aged 50–74 years [4]. In Europe, vaccination policies remain heterogeneous and implemented at the national level, with no harmonized EU-wide recommendation to date. In January 2026, the European Medicines Agency expanded the authorization of the GSK RSV vaccine (Arexvy, GlaxoSmithKline, Brentford, UK) to adults aged ≥18 years [32]. However, its incorporation into routine immunization programs is still in progress.

In this context, the absence of major differences in inflammatory responses between epidemic waves and the limited prognostic value of these biomarkers are consistent with a relevant role of host vulnerability in determining disease outcomes rather than differences in acute inflammatory activation. Given the substantial hospitalization burden observed in older adults and the risk of severe outcomes, our findings support strengthening preventive strategies, particularly vaccination in frail and elderly individuals, who represent the main risk group for severe disease.

Although multivariate adjustment would have accounted for potential confounding factors such as age, comorbidity burden, lifestyle factors, and disease severity, this was not feasible due to the limited number of outcome events and quasi-complete separation in the dataset, which prevented reliable convergence of the logistic regression models. Under these conditions, standard multivariate approaches yield unstable and non-interpretable estimates, a well-recognized limitation in small retrospective cohorts. As a result, residual confounding by factors such as age, comorbidity burden, or differences between hospitalized and non-hospitalized patients cannot be fully excluded. Nevertheless, the consistency of the findings across univariate analyses and of ROC curve performance supports the overall conclusion that the evaluated biomarkers do not show meaningful prognostic discrimination in this cohort.

Further limitations of this study should be acknowledged, including the retrospective single-center design and moderate sample size, which may limit the generalizability of the results. In addition, the absence of an external validation cohort increases the possibility of model overfitting and may have led to optimistic estimates of diagnostic performance. Moreover, using the McCabe score as a proxy for severity and dichotomizing outcomes may have reduced sensitivity for detecting subtle associations. In addition, virological variables such as RSV subtype, prior immunity, and vaccination status were not systematically available in the retrospective clinical records, and therefore could not be incorporated into the analyses. The use of pre-pandemic healthy controls may have led to overestimation of the evaluated biomarkers’ diagnostic performance, particularly IL-6 and the neutrophil-to-lymphocyte ratio. Since these markers reflect nonspecific systemic inflammatory activation, their discriminatory capacity would likely be lower in real-world clinical settings involving patients with other acute infectious or inflammatory conditions. In addition, no formal correction for multiple comparisons was applied due to the exploratory and descriptive nature of the study and the relatively limited sample size. Therefore, some statistically significant findings should be interpreted with caution due to the risk of Type I error inflation. Furthermore, no subgroup or stratified analyses according to admission status were performed because the number of non-hospitalized patients was limited, particularly during the second epidemic wave. Therefore, residual confounding related to baseline severity, comorbidity burden, and hospitalization status cannot be excluded. Finally, the restricted biomarker panel may not fully capture the complexity of the host immune response. Future multicenter studies with larger cohorts and independent validation sets are needed to confirm these findings and improve the robustness of risk-stratification models in adult RSV infection.

## 5. Conclusions

The results of this study highlight the importance of understanding RSV infection in hospitalized adults and provide a comparative characterization of two epidemic waves, showing a consistent systemic inflammatory response over time. This between-wave stability, together with the observed clinical outcomes, suggests that differences in patient baseline characteristics, rather than changes in inflammatory profiles, may better explain variations in disease presentation.

Inflammatory biomarkers demonstrated good diagnostic performance, particularly the neutrophil-to-lymphocyte ratio and IL-6, whereas their prognostic value for clinical outcomes was limited. Overall, these findings support a distinction between the diagnostic usefulness of inflammatory markers in acute infection and their limited ability to predict disease progression. These results underscore the relevance of host vulnerability and comorbidity burden in shaping outcomes, and support the need for preventive strategies in older at-risk adults.

## Figures and Tables

**Table 1:** Demographic and clinical characteristics of patients in the two waves of respiratory syncytial virus infection.

Variable	1st Wave (*n* = 81)	2nd Wave (*n* = 71)	*p* Value	Effect Size *
Demographic characteristics and coinfections				
Age, years	74 (22–96)	78 (20–96)	0.009	r = 0.21 (0.05–0.37)
Sex, female	43 (53.1)	39 (54.9)	0.820	OR = 0.93 (0.49–1.76)
Tobacco use	9 (11.1)	10 (14.1)	0.629	OR = 1.31 (0.50–3.43)
Alcohol drinking	12 (14.8)	5 (7.0)	0.196	OR = 0.44 (0.15–1.30)
Influenza A	2 (2.5)	3 (4.2)	0.545	OR = 1.74 (0.28–10.73)
Influenza B	0 (0.0)	0 (0.0)	1.000	Not calculated ^†^
COVID-19	0 (0.0)	0 (0.0)	1.000	Not calculated ^†^
Admissions department				
Geriatrics	8 (9.9)	20 (28.2)	<0.001	
Internal Medicine	29 (35.8)	38 (53.5)		
Emergency	37 (45.7)	9 (12.7)		
Oncology	4 (4.9)	3 (4.2)		Cramér’s V = 0.507
Surgery	0 (0.0)	1 (1.4)		
Direct ICU admissions	2 (2.5)	0 (0.0)		
Gynecology	1 (1.2)	0 (0.0)		
Ward-to-ICU transfers	5 (6.2)	5 (7.0)	0.829	OR = 1.15 (0.32–4.16
ICU length of stay, days	3 (2–10)	6 (5–11)	0.151	r = 0.33 (0.17–0.49)
Hospital length of stay, days	7 (1–35)	7 (1–29)	0.658	r = 0.05 (−0.11–0.21)
Symptoms				
Pneumonia	18 (22.2)	26 (36.6)	0.050	OR = 1.90 (0.93–3.89)
Bronchitis	16 (19.8)	46 (64.8)	<0.001	OR = 7.47 (3.59–15.54)
Cough	61 (75.3)	54 (76.1)	0.915	OR = 1.04 (0.49–2.19)
Fever	33 (40.7)	29 (40.8)	0.990	OR = 1.00 (0.52–1.92)
Odynophagia	3 (3.7)	3 (4.2)	0.869	OR = 1.15 (0.22–5.87)
Headache	2 (2.5)	4 (5.6)	0.317	OR = 2.36 (0.42–13.28)
Anorexia or hyporexia	2 (2.5)	4 (5.6)	0.317	OR = 2.36 (0.42–13.28)
Myalgia	16 (19.8)	2 (2.8)	0.001	OR = 0.12 (0.03–0.53)
Arthralgia	13 (16.0)	2 (2.8)	0.006	OR = 0.15 (0.03–0.70)
Pulmonary embolism	0 (0.0)	1 (1.4)	1.000	Not calculated ^†^
Other symptoms	45 (55.6)	61 (85.9)	<0.001	OR = 4.88 (2.19–10.86)
Comorbidities				
Diabetes mellitus	27 (33.3)	27 (38.0)	0.546	OR = 1.22 (0.63–2.39)
Cardiovascular disease	48 (59.3)	56 (78.9)	0.009	OR = 2.57 (1.25–5.28)
Chronic liver disease	3 (3.7)	3 (4.2)	0.869	OR = 1.15 (0.22–5.87)
Chronic lung disease	36 (44.4)	29 (40.8)	0.655	OR = 0.86 (0.45–1.65)
Chronic kidney disease	18 (22.2)	17 (23.9)	0.802	OR = 1.10 (0.52–2.35)
CNMD	14 (17.3)	18 (25.4)	0.238	OR = 1.63 (0.74–3.57)
Cancer	7 (8.6)	6 (8.5)	0.988	OR = 0.99 (0.32–3.10)
Charlson index				
0	25 (30.9)	17 (23.9)	0.191	
1	22 (27.2)	21 (29.6)		
2	15 (18.5)	23 (32.4)		
3	10 (12.3)	9 (12.7)		
4	5 (6.2)	1 (1.4)		Cramér’s V = 0.257
5	2 (2.5)	0 (0.0)		
6	1 (1.2)	0 (0.0)		
7	1 (1.2)	0 (0.0)		
McCabe score				
1	71 (87.7)	26 (36.6)	<0.001	
2	6 (7.4)	24 (33.8)		Cramér’s V = 0.548
3	4 (4.9)	21 (29.6)		
Treatments				
IMV	1 (1.2)	3 (4.2)	0.342	OR = 3.53 (0.36–34.72)
NIMV	3 (3.7)	11 (15.5)	0.022	OR = 4.71 (1.26–17.62)
Conventional oxygen therapy ^‡^	58 (71.6)	61 (85.9)	0.049	OR = 2.31 (1.01–5.30)
Anticoagulants	16 (19.8)	3 (4.2)	0.004	OR = 0.18 (0.05–0.64)
Corticosteroids	50 (61.7)	59 (83.1)	0.004	OR = 3.05 (1.42–6.55)
Deceased	11 (13.6)	8 (11.3)	0.668	OR = 0.81 (0.31–2.14)

Qualitative variables are presented as *n* (%), and quantitative variables as medians (minimum–maximum). Statistical comparisons were made using the χ^2^ test (categorical variables) or the Mann–Whitney U test (continuous variables). * Effect sizes were reported as rank-biserial correlations (r) for continuous variables, odds ratios (OR) with corresponding 95% confidence intervals for binary variables, and Cramér’s V for variables with more than two categories. All effect sizes were computed to complement the *p* values and to provide information on the magnitude of differences between groups. ^†^ Odds ratios were not calculated for variables for constant values of zero in at least one group. ^‡^ Conventional oxygen therapy refers to oxygen supplementation delivered via nasal cannula or Venturi mask. Abbreviations: CNMD, chronic neurological or neuromuscular disease; ICU, intensive care unit; IMV, invasive mechanical ventilation; NIMV, non-invasive mechanical ventilation.

**Table 2:** Effect size and median differences for biomarker comparisons between epidemic waves.

Variable	Effect Size (r)	Median Difference (95% CI)
Neutrophils	0.168	−1.26 (−2.44 to −0.08)
Lymphocytes	0.057	0.08 (−0.13 to 0.28)
N/L	0.138	−1.79 (−4.17 to 0.25)
IL-6	0.149	−0.26 (−0.55 to 0.04)
CRP	0.102	−0.12 (−0.32 to 0.07)

Effect sizes (r) were calculated using the Wilcoxon rank-sum test. Median differences (wave 1–wave 2) were estimated using the Hodges–Lehmann method with 95% confidence intervals. Abbreviations: CRP, C-reactive protein; IL-6, interleukin-6; N/L ratio, neutrophil-to-lymphocyte ratio.

**Table 3:** Receiver operating characteristic analysis of inflammatory biomarkers with bootstrap internal validation.

Group	Biomarker	AUC (95% CI)
First wave	IL-6	0.862 (0.779–0.931)
	CRP	0.754 (0.673–0.829)
	Neutrophils	0.866 (0.798–0.924)
	Lymphocytes	0.885 (0.824–0.938)
	N/L Ratio	0.892 (0.823–0.948)
Second wave	IL-6	0.863 (0.787–0.934)
	CRP	0.794 (0.716–0.856)
	Neutrophils	0.909 (0.852–0.960)
	Lymphocytes	0.901 (0.838–0.952)
	N/L Ratio	0.949 (0.907–0.985)

Values are expressed as area under the curve with 95% confidence intervals. Receiver operating characteristic analyses were performed separately for each epidemic wave, comparing each wave with the control group. Internal validation was conducted using bootstrap resampling (2000 iterations) to estimate confidence intervals. Abbreviations: AUC, area under the curve; CI, confidence interval; CRP, C-reactive protein; IL-6, interleukin-6; N/L ratio, neutrophil-to-lymphocyte ratio.

**Table 4:** Comparison of areas under the curve between epidemic waves.

Variable	AUC 1st Wave	AUC 2nd Wave	*p* Value
IL-6	0.862	0.863	0.983
CRP	0.754	0.794	0.461
Neutrophils	0.866	0.909	0.306
Lymphocytes	0.885	0.901	0.695
N/L Ratio	0.892	0.949	0.126

Values represent the area under the curve (AUC) for each biomarker in the first and second epidemic waves compared with the control group. Statistical comparisons between AUCs of the two epidemic waves were performed using the DeLong test. *p* values indicate differences between the first and second epidemic waves. Abbreviations: AUC, area under the curve; CRP, C-reactive protein; IL-6, interleukin-6; N/L ratio, neutrophil-to-lymphocyte ratio.

**Table 5:** Diagnostic performance, optimal cut-off values, and predictive values of the evaluated biomarkers.

Variable	AUC	Cut-off	Sensitivity	Specificity	PPV	NPV
IL-6	0.86 (0.81–0.92)	8.0 pg/mL	0.84 (0.77–0.89)	1.00 (0.96–1.00)	1.00 (0.97–1.00)	0.76 (0.69–0.82)
CRP	0.77 (0.71–0.83)	1.85 mg/dL	0.77 (0.69–0.83)	0.63 (0.51–0.73)	0.80 (0.74–0.84)	0.59 (0.51–0.67)
N	0.89 (0.84–0.93)	5.0 × 10^3^/µL	0.79 (0.72–0.85)	0.91 (0.83–0.96)	0.95 (0.87–0.99)	0.69 (0.62–0.78)
L	0.89 (0.85–0.94)	1.7 × 10^3^/µL	0.82 (0.74–0.87)	0.88 (0.78–0.94)	0.93 (0.87–0.96)	0.71 (0.64–0.78)
N/L	0.92 (0.89–0.96)	2.3	0.89 (0.83–0.93)	0.88 (0.78–0.94)	0.93 (0.88–0.96)	0.81 (0.72–0.87)

Data are expressed as point estimates and 95% confidence intervals (in parentheses). Optimal cut-off points were determined by maximizing the Youden index. Abbreviations: AUC, area under the curve; CRP, C-reactive protein; IL-6, interleukin 6; L, lymphocyte count; N, neutrophil count; N/L ratio, neutrophil-to-lymphocyte ratio; NPV, negative predictive value; PPV, positive predictive value.

**Table 6:** Effect size estimates for biomarker comparisons according to McCabe-defined severity and mortality.

Variable	McCabe (r)	Interpretation	Mortality (r)	Interpretation
IL-6	0.077	small	0.066	small
CRP	0.059	small	0.068	small
Neutrophils	0.070	small	0.076	small
Lymphocytes	0.130	small	0.099	small
N/L Ratio	0.105	small	0.033	small

Effect sizes (r) were calculated using the Wilcoxon rank-sum test. Interpretation is based on standard thresholds: negligible (<0.1), small (0.1–0.3), moderate (0.3–0.5), and large (>0.5). Sample sizes for each subgroup are indicated in the table. Hodges–Lehmann estimates were not calculated for subgroup analyses due to small sample sizes in some strata. Abbreviations: CRP, C-reactive protein; IL-6, interleukin-6; N/L ratio, neutrophil-to-lymphocyte ratio.

## Data Availability

The data presented in this study are available on request from the corresponding author due to privacy and confidentiality constraints.

## Abbreviations

The following abbreviations are used in this manuscript:

AUC	Area under the curve
CRP	C-reactive protein
ICU	Intensive care unit
IL-6	Interleukin-6
N/L ratio	Neutrophil-to-lymphocyte ratio
NPV	Negative predictive value
PPV	Positive predictive value
ROC	Receiver operating characteristic
RSV	Respiratory syncytial virus
